# Patient report outcomes in cryoballoon ablation of atrial fibrillation during the COVID Era: Insights from the 1STOP project

**DOI:** 10.1007/s10840-023-01561-5

**Published:** 2023-05-13

**Authors:** Giulio Molon, Giuseppe Arena, Claudio Tondo, Danilo Ricciardi, Pietro Rossi, Paolo Pieragnoli, Roberto Verlato, Massimiliano Manfrin, Giulia Girardengo, Giuseppe Campisi, Domenico Pecora, Mario Luzi, Saverio Iacopino

**Affiliations:** 1 IRCCS Sacro Cuore Don Calabria Hospital, Negrar, Verona, Italy; 2 Ospedale delle Apuane, Massa, Italy; 3 Centro Cardiologico Monzino, IRCCS Department of Electrophysiology&Cardiac Pacing Department of Biomedical, Surgery and Dentist Sciences, University of Milan, Milan, Italy; 4 Campus Biomedico, Rome, Italy; 5 Fatebenefratelli, Rome, Italy; 6 ULSS 6 Euganea, Camposampiero-Cittadella, Italy; 7 Ospedale Careggi, University of Florence, Firenze, Italy; 8 Ospedale San Maurizio, Bolzano, Italy; 9 Ospedale San Luca, IRCCS Istituto Auxologico, Milano, Italy; 10 PO Giovanni Paolo II, Ragusa, Italy; 11 Poliambulanza, Brescia, Italy; 12 Ospedale Provinciale, Macerata, Italy; 13 Maria Cecilia Hospital, GVM Care & Research, Cotignola, Italy

**Keywords:** Atrial fibrillation, cryoballoon, outcomes, AF recurrences, Patient Reported Oucomes, AF related symptoms

## Abstract

**Background:**

Pulmonary vein isolation by cryoablation (PVI-C) is a standard therapy for the treatment of patients with symptomatic atrial fibrillation (AF). AF symptoms are highly subjective; however, they are important outcomes for the patient. The aim is to describe the use and impact of a web-based App to collect AF-related symptoms in a population of patients who underwent PVI-C in seven Italian centers.

**Methods:**

A patient App to collect AF-related symptoms and general health status was proposed to all patients who underwent an index PVI-C. Patients were divided into two groups according to the utilization of the App or the non-usage.

**Results:**

Out of 865 patients, 353 (41%) subjects composed the App group, and 512 (59%) composed the No-App group. Baseline characteristics were comparable between the two cohorts except for age, sex, type of AF, and body mass index. During a mean follow-up of 7.9±13.8 months, AF recurrence was found in 57/865 (7%) subjects with an annual rate of 7.36% (95% CI:5.67-9.55%) in the No-App versus 10.99% (95% CI:9.67-12.48%) in the App group, *p*=0.007. In total, 14,458 diaries were sent by the 353 subjects in the App group and 77.1% reported a good health status and no symptoms. In only 518 diaries (3.6%), the patients reported a bad health status, and bad health status was an independent parameter of AF recurrence during follow-up.

**Conclusions:**

The use of a web App to record AF-related symptoms was feasible and effective. Additionally, a bad health status reporting in the App was associated with AF recurrence during follow-up.

**Supplementary Information:**

The online version contains supplementary material available at 10.1007/s10840-023-01561-5.

## Introduction

To date, the electrical isolation of the pulmonary veins (PVs) is a class I indication in patients that are refractory or intolerant to at least one Class I or III antiarrhythmic medication with recurrent symptomatic atrial fibrillation (AF) [1, 3]. Patients’ experience of AF symptoms and their management are highly subjective and can change not only from patient-to-patient, but also, before and after catheter ablation of AF. Nevertheless, measuring outcomes that are important to patients, including symptoms in addition to clinical endpoints (*e.g.,* AF recurrence, death, and stroke), can improve AF management and care. In brief, the patient experience is an important assessment for success of the medical treatment from the patient’s perspective [1]. However, healthcare systems and therapies are only starting these patient-centric and holistic approaches to care.

Consequently, health informatics systems, smartphones, and remote web Apps can help to capture patient reported outcome (PRO) data, in particular, during the COVID-19 pandemic period when several restrictions aimed at containing the spreading of infection limited patients’ access to healthcare services (*e.g.,* routine follow-up and office visits) [4, 5]. In our analysis, we described the use and the impact of a web-based App to collect AF-related symptoms in a population of patients who underwent cryoballoon ablation in the setting of a real-word experience within Italian centers.

## Methods

### Project design & MYCRYO APP

Consecutive patients with paroxysmal or persistent AF who underwent an index pulmonary vein isolation (PVI) using a cryoballoon catheter (Artic Front Advance; Medtronic, Inc.) from May 2020 until December 2021 in seven Italian centers (participating to the One Shot TO Pulmonary vein isolation (1STOP) ClinicalService project using MYCRYO APP) were included in the analysis. ClinicalService is a national cardiovascular data repository and medical care project designed to describe and improve the quality of diagnostic and therapeutic strategies using technologies and therapies in the Italian clinical practice [6, 7]. The project consists of a shared environment for the prospective collection, management, analysis, and reporting of data from patients in whom Medtronic therapies have been applied.

A new smartphone application, MYCRYOAPP, was used in this pivotal experience to pool PRO data with clinical data in the One Hospital ClinicalService environment. Based upon the patient’s AF diagnosis/treatment and their own decision to use the MYCRYOAPP; subjects that were evaluated for this analysis had the following criteria, including: 1) the patient had drug-refractory and symptomatic AF; 2) the patient had elected to undergo catheter ablation of AF by cryoballoon PVI; and 3) the patient was able to download the MYCRYOAPP unto their own communication device (*e.g.,* an android or IOS smartphone).

**Fig. 1:**
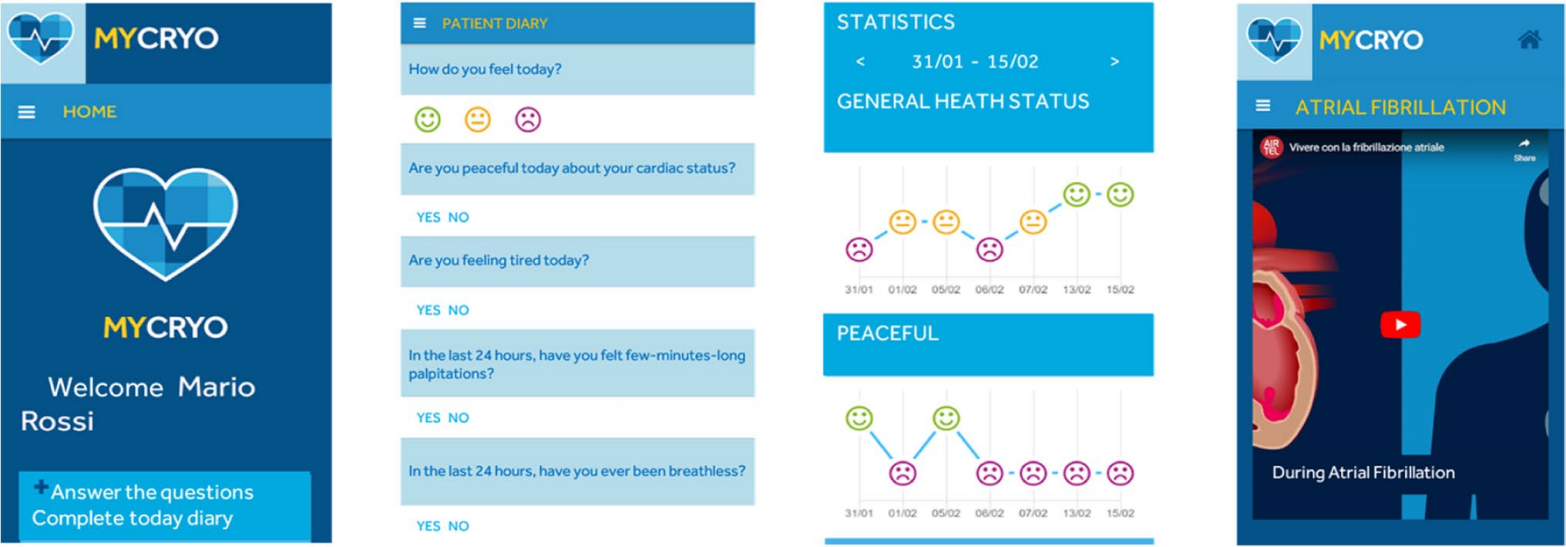
Screenshot of MYCRYO APP, a smartphone application for patients treated by ablation therapies for atrial fibrillation

The patients were requested to periodically answer up to five simple questions about their AF-related symptoms and their general health status, including: 1) How do you feel today? 2) Today, regarding your illness, do you feel peaceful? 3) Have you experienced fatigue and weakness, today? 4) Have you experienced palpitations lasting a few minutes in the last 24 hours? and 5) Have you experienced dyspnea in the last 24 hours? As depicted in Fig. 1, patient responses were tracked daily by either a “yes or no” response or facial “mood” emoji depending on the question. Moreover, within the App (in a dedicated section), patients can find more information on AF disease and therapies, and in particular, there are videos and brochure-styled materials that explain the AF disease and process, the main causes of AF, the effects of AF on heart rate, and general information on patient’s health. Additionally, these multimedia tools provide patient education materials in an “easy-to-understand” format which includes information on the catheter ablation procedure as well as arrhythmia-control medications. Also, patients can review their personal trends including their own answers on AF burden from the previous three months to further understand their own disease symptom(s). Importantly, the physician can review all the patient’s questionnaires and the trend of the answers in an internet-accessible website.

In this unique experience, this novel study design used the patient’s answers and trends as a patient’s diary, to collect the patient’s health status and AF-related symptoms. Patients were asked to complete an App questionnaire at least once per week if they were asymptomatic, and otherwise, they were asked to report all AF-related symptoms during follow-up through the usage of the App. The trend(s) of symptom(s) were reviewed by each physician either in real-time or during an in-person clinic follow-up, according to the clinical practice of each center. Additionally, the physicians could download from the website a weekly report to show the patients’ compliance to identify non-compliant patients. Every week the App sent a notification to non-compliant patients in order to encourage them to fill out the questionnaire and re-engage with the App.

This project was approved by each site’s Institutional Review Board and Local Ethics Committees and conforms to the principles outlined in the 1975 Declaration of Helsinki as reflected in the *a priori* approval by the institution's human research committee. Each patient included in the ClinicalService project provided informed consent for data collection and analysis [6, 7].

The primary objective of this analysis was to describe the patients who accepted the usage of a web-based App as a diary to collect AF-related symptoms. Additionally, this analysis did assess the usage and utility of this App-based technology in patients with AF. Specifically, this analysis evaluated the impact of AF-related symptoms declared by the patients on AF recurrence during follow-up (after the index ablation). The population was divided into two groups. The App group were the patients who were proposed the MYCRYOAPP and who then used the App. Conversely, the No App group were the patients followed with standard follow-up visits according to the clinical practice of each center. The reasons for the failure to use the App were mainly two, including: 1) the patients declined the use of the App, and/or 2) the lack of hospital resources/committed nurses to train the patients on the downloading of the App and on the use of the App during the COVID-19 Pandemic.

### Population and procedural characteristics

During the baseline visit, several patient clinical characteristics and histories were collected, including age, sex, date of first AF diagnosis, NYHA class, CHA_2_DS_2_-VASc score, previous antiarrhythmic drug therapy, hypertension status, and previous thromboembolic events. During the PVI-C procedure, we collected data on procedure duration (skin-to-skin), fluoroscopy time, and the overall ablation time. All information about any adverse events occurring during the procedure were recorded and collected, including permanent and transient diaphragmatic paralysis, pericarditis, arteriosus-venous fistula, cardiac tamponade, transient ischemic attack (TIA), stroke, and other minor complications. During the pre-discharge discussion, the MYCRYOAPP was provided to the patient (in accordance with the patient’s decision). Patients and/or their families were instructed on how to download and use the App by a staff of trained and committed nurses. A primary focus of the App training session was to relay the importance of compliance to reporting AF-related symptoms for the patient and to complete the questionnaire at least once per week or when symptom(s) occurred.

### Follow-up and event collection

Regardless the usage of MYCRYOAPP, follow-up visits were made in accordance with the clinical practice of each center, including clinic visits every three months (during the first year after the index PVI-C) and clinic visits every six months, thereafter. Due to COVID-19 restrictions, some office visits were performed remotely or postponed during the first year of follow-up. The standard visit consisted of an assessment of the patient’s AF-related symptoms, an ECG or Holter monitoring examination, and an assessment of the patient’s pharmaceutical medications. In the App group, the trends of the PROs, the symptoms, and health status were analyzed using the web site and matched with all other patient data. To minimize the number of non-compliant patients, automatic notifications were sent to the patient when a diary was not received at least once per week to remind them to complete the diary. During this pilot experience, although the MYCRYOAPP was updated in real-time, patient symptoms and trends were only displayed and analyzed during the remote or in-person follow-up. The first 90 days after PVI-C were denoted as a landmark “90-day blanking period” whereby no recurrence of AF was counted against the primary efficacy endpoint [1–3]. Since antiarrhythmic drug (AAD) usage following PVI-C was performed according to each center’s practice (rather than a standardized protocol); we analyzed the recurrence of atrial arrhythmia, defined as an electrocardiographic documented episode of AF or atrial tachycardia lasting at least 30 seconds after the performance of an index PVI-C procedure, in the entire population regardless of the usage of AADs after the 90-day blanking period [6, 7].

### Statistical analysis

Descriptive statistics were used to summarize patient characteristics. These include mean and standard deviation, minimum, maximum, and median with the interquartile range (IQR) for continuous variables. Counts and percentages were used for categorical variables. Summary statistics were reported with a maximum of two decimals, as appropriate. Comparisons between groups were performed using the Wilcoxon’s Test for continuous variables, while comparisons of categorical variables were performed using the Chi-square test or Fisher’s exact test for extreme proportions, as appropriate. When comparing data from diaries, a GEE model was used to consider the within-patient correlation. Incidence Rates (IRs) were expressed as number of events / 100 patient-years, and estimated using Poisson regression models, with deviance scaling to correct for over/under dispersion. Estimates along with their 95% Confidence Intervals (CIs) were reported. Estimated differences between groups were expressed as Incidence Rate Ratios (IRRs), along with their 95% CIs. To find predictors of AF recurrences, a logistic regression was used for both univariate and multivariate analyses, and the proportional hazard hypothesis was tested. A set of *a priori* potential predictors were assessed. Possible collinearity among these variables were tested by Spearman-Rho, where a correlation coefficient >0.80 and/or clinical judgment determined covariate exclusion. The final set of potential predictors was then included in the multivariate model. The Odds Ratios (ORs) and 95% confidence intervals (95% CIs) were estimated for all initial potential predictors. Statistical tests are based on a two-sided significance level of 0.05. The SAS software, version 9.4, (SAS Institute Inc., Cary, NC, USA) was used to perform statistical analyses.

## Results

In this analysis, 865 consecutive subjects with paroxysmal and persistent AF underwent an index PVI-C strategy of ablation. Of which, 353 (41%) subjects composed the App group, and 512 (59%) subjects composed the No App group (Supplementary Figure 1).

### Baseline and Procedural Characteristics

Baseline patient characteristics and procedural data are listed in Table 1. There were few differences between the App and No App group with regard to baseline characteristics. The App group was more likely young, male, had a higher BMI, and paroxysmal AF. All other baseline characteristics did not differ, including LA dimensions, CHA_2_D_2_-Vasc score, history of stroke/TIA, or hypertension. The mean total procedural, fluoroscopy, and ablation times were 85.6 ± 47.2, 25.8 ± 17.9, and 15.8 ± 4.9 minutes, respectively. No difference between the two groups was observed in the rate of acute complication which occurred in 19 patients (2.2%; Table 2).

**Table 1:** Baseline characteristics of the total population and statistical comparisons between the two groups of patients: subjects in the No APP group versus the App group

Baseline Characteristics	TOTAL (*n*=865)	No App Group (*n*=512)	App Group (*n*=353)	*p*-value
Age at first ablation (yrs)	58.2 ± 11.8	58.7 ± 13.0	57.5 ± 9.9	0.003
Gender (Female)	27.6% (239)	31.1% (159)	22.7% (80)	0.007
BMI (Kg/m^2^)	27.2 ± 4.4	26.8 ± 4.3	27.7 ± 4.5	0.014
Persistent Atrial fibrillation	33.1% (286)	36.1% (185)	28.6% (101)	0.021
Paroxysmal Atrial fibrillation	66.9% (579)	63.9% (327)	71.4% (252)	
Months from first Atrial Arrh. Episode to Ablation	52.4 ± 63.9	54.0 ± 68.9	50.3 ± 56.7	0.691
Number of tested AAD 2+	26.8% (231)	26.5% (135)	27.2% (96)	0.851
EHRA (mean±SD )	1.9 ± 0.7	1.9 ± 0.6	1.9 ± 0.8	0.976
History of Stroke/TIA	4.1% (35)	3.4% (17)	5.0% (18)	0.265
Cardiac Insufficiency	2.3% (19)	1.3% (6)	3.7% (13)	0.034
Hypertension	46.4% (401)	44.2% (226)	49.4% (175)	0.147
Any Valve disease	5.3% (45)	4.7% (24)	6.1% (21)	0.402
CHA_2_DS_2_-VASc				
0	31.2% (270)	30.9% (158)	31.7% (112)	0.757
1	31.8% (275)	30.3% (155)	34.0% (120)	
2	21.5% (186)	22.1% (113)	20.7% (73)	
3	11.8% (102)	12.9% (66)	10.2% (36)	
4	3.0% (26)	3.1% (16)	2.8% (10)	
≥5	0.7% (6)	0.8% (4)	0.6% (2)	
Diabetes	8.8% (76/865)	8.9% (45/512)	8.6% (31/353)	0.867
CKD	1.9% (16)	1.6% (8)	2.3% (8)	0.494
Left Ventricle Ejection Fraction	58.6 ± 7.4	58.8 ± 7.3	58.4 ± 7.6	0.373
Left Atrial Diameter	39.9 ± 6.9	39.6 ± 6.6	40.1 ± 7.0	0.474
Class III Anti-arrhythmic Drugs at baseline	23.0% (198)	22.5% (115)	23.7% (83)	0.704
Class I Anti-arrhythmic Drugs at baseline	40.7% (352)	38.8% (198)	43.3% (154)	0.216
Class III Anti-arrhythmic Drugs post Blanking period	6.6% (57)	5.8% (30)	7.6% (27)	0.723
Class I Anti-arrhythmic Drugs post Blanking period	16.4% (142)	17.1% (88)	15.2% (54)	0.432

*AAD* anti-arrhythmic drug, *TIA* transient ischemic attack, *CKD* Chronic Kidney disease

**Table 2:** Procedural times and periprocedural complications of the total population and comparison between the two groups of patients: subjects in the No APP group versus the App group

Procedural Characteristics	TOTAL (*n*=865)	No App Group (*n*=512)	App Group (*n*=353)	*p*-value
Procedure Duration (min)	75.0 (60.0 - 100.0)	75.0 (60.0 - 93.0)	80.0 (60.0 - 115.0)	0.002
Fluoroscopy Duration (min)	21.0 (13.9 - 32.0)	19.2 (12.0 - 30.0)	23.7 (15.5 - 37.0)	<0.001
Ablation time (min)	16.0 (12.0 - 18.0)	15.5 (12.0 - 16.0)	16.0 (13.0 - 20.0)	<0.001
Patients with at least one complication	2.2% (19/865)	2.0% (10/512)	2.5% (9/353)	0.556
Permanent Diaphragmatic Paralysis	0.1% (1/865)	0.0% (0/512)	0.3% (1/353)	0.408
Transient Diaphragmatic Paralysis	1.6% (14/865)	1.8% (9/512)	1.4% (5/353)	0.696
Peri-cardiac effusion	0.2% (2/865)	0.2% (1/512)	0.3% (1/353)	1.000
Femoral pseudo-aneurism	0.1% (1/865)	0.0% (0/512)	0.3% (1/353)	0.408
Other complication	0.2% (2/865)	0.0% (0/512)	0.6% (2/353)	0.166

### AF Recurrence

No subject was lost to follow-up, and the mean follow-up period was 7.9 ± 13.8 months. AF recurrence was found in 57/865 (7%) subjects, and the annual rate of AF recurrence was 7.36% (95% CI: 5.67-9.55 %) in the No App group compared to 10.99% (95% CI: 9.67-12.48 %) in the App group, p=0.007.

### MYCRYO APP Usage

In total, during the follow-up, 14,458 diaries were sent by 353 subjects during the follow-up period. On average, each patient completed 41.7 ± 94.1 diaries. Out of 14,458 diaries, 11,150 (77.1%) reported a good general health status and no AF related symptoms, while in 2,790 diaries (19.3%) the general health status was “so-so.” In only 518 diaries (3.6%), the patients reported a bad health status. In Table 3, the most frequent recorded symptom was tiredness in 14.0% (2020/14458) of surveys, then palpitations in 12.0% (1729/14458), and lack of breath in 5.2% (749/14458) of the reported surveys to the App. To assess the impact of reported symptoms, a sub-analysis in the App group was designed comparing the diaries between the subjects with AF recurrence (30) to those without AF recurrence (323) after the blanking period. Supplementary Table 1 shows the number of diaries in the group of patients with and without AF recurrence. Of the total 353 aforementioned patient, 46.6% of patients having AF recurrence reported a “bad” heath status as a daily feeling in 66/1300 (5.1%) questionnaires as compared 24.8% in 144/ 8545 (1.7%) without AF recurrence, p<0.001 (Table 4). Considering the answer “bad” as a predictor of AF recurrence (at multivariate analysis, OR: 2.64, 95% CI: 1.20-5.79, p=0.016), the findings determined a specificity of 75% and a negative predictive value of 90% (Supplementary Table 2).

**Table 3:** Summary of 14458 diaries collected in 353 patients during the follow up

Diaries Parameter	TOTAL (*n*=14458)
Daily Feeling	
Good	77.1% (11150/14458)
So and So	19.3% (2790/14458)
Bad	3.6% (518/14458)
Perceived health status	
Cardiac condition – no issues	88.9% (12851/14458)
Symptoms	
Tiredness	14.0% (2020/14458)
Palpitations	12.0% (1729/14458)
Lack of breath	5.2% (749/14458)
Number of symptoms recorded	
0	77.2% (11165/14458)
1	16.3% (2353/14458)
2	4.7% (675/14458)
3	1.8% (265/14458)

**Table 4:** Summary of 9845 diaries collected in 353 patients during the follow up after the blanking period (3 months after the PVI procedure) according to the presence of AF recurrences

Diaries Parameter	TOTAL (*n*=9845)	Diaries of Patients without AF Recurrence (*n*=8545)	Diaries of Patients with AF Recurrence (*n*=1300)	*p*-value*
Daily Feeling				
Good	75.7% (7453/ 9845)	78.0% (6668/ 8545)	60.4% (785/ 1300)	<0.001
So and So	22.2% (2182/ 9845)	20.3% (1733/ 8545)	34.5% (449/ 1300)	
Bad	2.1% (210/ 9845)	1.7% (144/ 8545)	5.1% (66/ 1300)	
Cardiac condition – no issues	86.9% (8556/ 9845)	90.2% (7705/ 8545)	65.5% (851/ 1300)	<0.001
Tiredness	15.2% (1499/ 9845)	15.6% (1329/ 8545)	13.1% (170/ 1300)	0.021
Palpitations	12.0% (1185/ 9845)	11.6% (987/ 8545)	15.2% (198/ 1300)	<0.001
Lack of breath	5.4% (536/ 9845)	5.0% (429/ 8545)	8.2% (107/ 1300)	<0.001
Maximum number of symptoms recorded				
0	76.0% (7484/ 9845)	76.3% (6518/ 8545)	74.3% (966/ 1300)	0.121
1	17.1% (1682/ 9845)	17.0% (1454/ 8545)	17.5% (228/ 1300)	0.641
2	5.1% (499/ 9845)	5.0% (428/ 8545)	5.5% (71/ 1300)	0.488
3	1.8% (180/ 9845)	1.7% (145/ 8545)	2.7% (35/ 1300)	0.013
2+	6.9% (679/ 9845)	6.7% (573/ 8545)	8.2% (106/ 1300)	0.055

## Discussion

### Main results

Individualized medicine and patient-centered care together with patient’s empowerment are three important factors of contemporary healthcare [1, 8–11]. In particular, in the treatment of recurrent and symptomatic AF, knowing the outcomes perceived by the patient and their relations with clinical outcomes may improve the care and the management of patients during follow-up clinical care. This current analysis demonstrated that (of 865 patients, 353 subjects in the App group and 512 in the No-App group) AF recurrence was found in 57/865 subjects with an annual rate 10.99% in the App group versus 7.36% in the No-App group which was statistically different. In total, 14,458 diaries were sent by 353 subjects in the App group and 77.1% reported a good health status and no AF-related symptoms. Conversely, the patients reported a bad health status in 3.6% of diaries, and bad health status was an independent predictor of AF recurrence during follow-up.

Some pilot study experiences in collecting PRO in patients with AF have been published [12, 13]. Steinberg *et al.* described a systematic AF PRO implementation in a tertiary-care electrophysiology clinic, assessing the feasibility of routine collection in the clinical practice deploying the questionnaires via electronic e-mail invitation to selected patients undergoing catheter ablation for AF [13]. In our pivotal experience, the patients were invited to the centers to download an App onto their own smartphones and to answer to only five simple questions in order to collect the general health status and the presence of more common AF-related symptoms. The ICHOM working group proposed a standard set of patient-reported outcomes including these sub-domains (*i.e.,* Quality of Life, emotional functioning, physical functioning, exercise tolerance, symptom severity, ability to work, and cognitive functioning) [14].

The capability to collect and measure outcomes of patients in a standardized manner and in the clinical practice is, on one hand, the key to improving the potential of value-based healthcare and to realizing a patient–centric approach towards medicine. However, new challenges related to hospital organizations, time consuming activities, and dedicated/trained personnel must be considered when viewing these novel patient approaches across the healthcare landscape (inclusive of economic costs and value). The use of an automatic App may be a practical method to reduce the burden on the healthcare personnel’s time; however, the full impact on the healthcare system still needs to be assessed. A cautionary example being the vast amount of ECGs that now have to be medically reviewed and archived as more patients monitor cardiac function (and in particular arrhythmia) through smartphone Apps. While these patient-centric approaches are novel and valuable, there is a need to balance the workload efforts with more traditional routes of care.

Moreover, the App could be provided to patients before the ablation procedure. This approach may facilitate better clinical patient diagnosis and management, including: 1) the measurement of frequencies of symptoms before the ablation procedure may help in stratifying patients; 2) a comparison of patient symptoms before and after the ablation procedure may facilitate a patient centric continued care guidance/metric; and 3) an ability to interact with patients before an ablation procedure (inclusive of links to find more information about the cardiac disease and treatment options) may drive more patient engagement and compliance to continued medical care. In our pilot study (presented here), we decided to use the App as a patient diary to detect AF-related symptoms in a more objective manner. For this reason, the presence and frequency of the symptoms were analyzed for only the time period following the ablation procedure. Nevertheless, the data were available in real time, and consequently, there was an opportunity to intervene and contact the patient immediately. Importantly, further studies are needed to understand which approach is the best and sustainable methodology.

In our pilot experience, the patients who accepted the usage of the App were more likely to be younger, to be men, and to have paroxysmal AF. Patients “empowerment and engagement” in collecting symptoms may also facilitate their disease management. Hussein *et al.* developed a fully automated platform to collect PRO and evaluate its first clinical application in a prospective cohort of AF ablations [15]. The study observed an increasing number of follow-up assessments and duration of follow-up, compared with routine care alone [15]. In our study experience, the patients were requested to use the App as a diary. Consequently, during the follow-up, the EP was able to match physical examinations, drug assessments, and clinical exams with the trend(s) of the reported symptoms.

Importantly, we observed that in patients with AF recurrence, about half of the patients (46.5%) reported (in the daily diary) the answer “bad” as a feeling compared with 24.5 % of patients without AF recurrence (p=0.012). Moreover, “bad” daily feeling resulted an independent predictor of AF recurrence. It is surprisingly that the feeling “bad” status correlated with AF recurrence rather than cardiac symptoms, including palpitation. One of the reasons could be that after catheter ablation the patients could perceive symptoms differently. In particular, the perception of palpitations can change over the time [16]. Also, palpitation can be caused by extra beats rather than AF, which is consistent with an overall reduction in AF-related symptom burden in those patients still experiencing general health symptoms. Our 1STOP Italian experience of patient management through the patient App started and had been conducted during the COVID-19 pandemic period. In Italy, since March 2020, severe restriction rules on mobility have been imposed to all citizens, and the whole healthcare national system was under an incredibly stress. For several months during 2020 and 2021, hospitals and medical centers had been dedicated to the care of COVID-19 patients, with cancellation of every non-urgent medical and surgical activities. Consequently, there was a loss of follow-up visits for patients with chronic heart diseases [17–19]. During the pandemic period, we have also witnessed the increase of AF burden, in non-infected people, as shown by the experience on patients with cardiac implantable electronic device (CIEDs) [20, 21], and in infected people hospitalized with COVID-19 [22]. The use of new technologies, such as telemedicine, had mitigated the lack of outpatient visits, especially in people with CIEDs [20, 21, 23].

However, all remote technologies (including MYCRYO APP) born, enhanced, or increased to meet the needs of social distancing, continue to be appreciated and used amongst our patients. In particular, nowadays, the remote technologies have become part of the clinical routine and have been integrated with traditional follow-up. Ultimately, our experience and the results may be influenced by the COVID-19 pandemic era, but we think that the experience made during this pandemic may improve patient management through the use of unique remote devices and services. The use of MYCRYOAPP in this study has been shown to detect clinical recurrences of AF to an even significantly greater extent than the usual management without the use of the App (10.99% vs 7.36% - p=0.007), thus showing that it can make an important contribution precisely in the COVID-19 era. To our knowledge, we are not aware of other experiences using Apps during the COVID-19 pandemic period to monitor patients with heart diseases, but we consider that these technologies could be useful for this purpose of monitoring. In fact, the use of the App-based technology to collect symptoms has involved patients much more in the care of their own health and particularly in the management of their recurrences of AF, allowing a significant greater diagnostic power in regard to patients with standard follow-up.

Importantly, the bad feeling had a very high negative predictive value that could help in managing frequency and type of follow-up, especially during COVID-19 and the post-COVID pandemic time which we are currently treating. Moreover, the App-based technology could be implemented in the daily clinical practice in case of compliant patients to improve the quality of follow-ups collecting patient reported outcomes and managing the frequency of clinical examination, including ECGs, Holter monitoring, or/and drug therapies. Further randomized studies are needed to assess the impact of these new tools in the clinical practice.

## Limitations

This analysis presents some limitations, including it was a non-randomized project, so bias could be present in the patient selection and treatment, and the patient App was proposed to consecutive patients who underwent PVI ablation in the participating centers. Nevertheless, we cannot exclude bias in patient selection and App usage. In particular, selection bias may have affected the data set as it is possible that there were socioeconomic factors (e.g., education, family support, etc.) which influenced which patients favored the App usage. A randomize clinical trial is needed to assess the value of the patient App in collecting symptoms and measuring the impact of the App on AF recurrence detection and patient management.

No data on education of patients or the reasons for declining were collected. Data were based on the clinical practice of several participating centers with different standard-of-care procedures. As per the nature of this project, follow-up assessments were made in accordance with the standard clinical practices at each center. No protocol was shared amongst the centers. Nevertheless, during a standard follow-up clinic visit, each center completed an ECG exam, a Holter examination (if present), and an assessment/review of current drug therapy.

During the patient App collect, there were only five simple questions and no validated Quality of Life (QoL) questionnaires. App questions were selected by a pool of physicians but were not validated in any previous study. The usage of validated questions may have improved the efficacy of the App. However, the strength of this data set resides in the number and variety of participating centers.

A further limitation is that the study had been conducted during the COVID-19 pandemic period. Consequently, some in-person visits could have been replaced by remote visits or canceled. This may under-estimate the rate of AF recurrence detection in both groups. Moreover, the pandemic period could have influenced the results presented in this study. Further studies are needed to show the benefit of patient Apps integrated in the standard follow-up during a post-pandemic period. Lastly, the follow-up of this analysis is short-term, and more data on long-term outcomes are needed to better assess the efficacy and utility of the App.

## Conclusions

In our real-world pivotal experience, the use of a patient App to collect health status and general symptoms was feasible and successfully. A “bad” status reporting was a predictor of AF recurrence during the follow-up after an index PVI-C.

## Supplementary Information

ESM 1: Supplementary Table 1: Patient APP usage in the whole patient population with APP and according to the presence of AF recurrences. Supplementary Table 2: Univariate and Multivariate analysis to assess the correlation between the Bad daily status and AF recurrence. Supplementary Figure 1. Flow chart of the patient population
