# Supplementary material for: Patient report outcomes in cryoballoon ablation of atrial fibrillation during the COVID Era: Insights from the 1STOP project
Source: J Interv Card Electrophysiol. 2023 May 13;67(1):61–9. doi: 10.1007/s10840-023-01561-5 (PMC10182349; doi:10.1007/s10840-023-01561-5)
Supplement: Supplementary file 1 — Supplementary Table 1: Patient APP usage in the whole patient population with APP and according to the presence of AF recurrences. Supplementary Table 2: Univariate and Multivariate analysis to assess the correlation between the Bad daily status and AF recurrence. Supplementary Figure 1. Flow chart of the patient population [file 10840_2023_1561_MOESM1_ESM.docx]

Supplementary material

Supplementary Table 1: Patient APP usage in the whole patient population with APP and according to the presence of AF recurrences

| ***App Parameter*** | ***TOTAL***  ***(n=353)*** | ***No AF recurrence***  ***(n=307)*** | ***AF recurrence***  ***(n=46)*** | ***p-value*** |
| --- | --- | --- | --- | --- |
| Age at first ablation (yrs) | 57.5 ± 9.9 | 57.4 ± 9.8 | 58.2 ± 11.0 | 0.296 |
| Number of recorded Diaries per patient | 41.7 ± 94.1 | 41.5 ± 94.5 | 43.3 ± 92.3 | 0.534 |
| Maximum number of recorded consecutive Diaries per patient | 2.0 (1.0 - 5.0) | 2.0 (1.0 - 5.0) | 2.0 (1.0 - 6.0) | 0.992 |

Supplementary Table 2: Univariate and Multivariate analysis to assess the correlation between the Bad daily status and AF recurrence.

| **Univariate Analysis** | | | **Multivariate Analysis** | |  |
| --- | --- | --- | --- | --- | --- |
| **Parameter** | **Odds Ratio (OR) (95% CI)** | **p-value** | **Odds Ratio (OR) (95% CI)** | **p-value** | |
| Age (continuous) | 0.99 (0.96-1.03) | 0.775 | 0.99 (0.96-1.03) | 0.769 | |
| Male gender | 0.84 (0.34-2.09) | 0.710 | 0.90 (0.35-2.32) | 0.832 | |
| Bad Daily Feeling | 2.66 (1.21-5.82) | 0.014 | 2.64 (1.20-5.79) | 0.016 | |
| Palpitations | 1.26 (0.58-2.72) | 0.560 | 1.22 (0.56-2.68) | 0.615 | |
| Lack of breath | 1.86 (0.86-4.03) | 0.118 | 1.83 (0.83-4.03) | 0.135 | |
| Tiredness | 1.13 (0.78-1.18) | 0.578 | 1.10 (0.75-1.18) | 0.589 | |
| At least two symptoms | 2.18 (1.00-4.76) | 0.051 | 2.16 (0.97-4.78) | 0.058 | |

Supplementary Figure 1. Flow chart of the patient population
